# Maxillary Frenulum and “Lip Tie”: What Parents Understand

**DOI:** 10.1002/oto2.71

**Published:** 2023-09-05

**Authors:** Beatrice R. Bacon, Michele M. Carr

**Affiliations:** ^1^ Jacobs School of Medicine and Biomedical Sciences University at Buffalo Buffalo New York USA; ^2^ Department of Otolaryngology, Jacobs School of Medicine and Biomedical Sciences University at Buffalo Buffalo New York USA

**Keywords:** breastfeeding, frenotomy, social media, superior labial frenulum

## Abstract

**Objective:**

To determine the proportion of parents that have some knowledge of abnormal maxillary frenulum, or “lip tie,” and their sources of this information.

**Study Design:**

Cross‐sectional study.

**Setting:**

Otolaryngology clinic.

**Methods:**

Consecutive parents of children ≤12 years of age presenting at a pediatric otolaryngology clinic were surveyed to discover their understanding of “lip tie” in children. The survey included questions on the effects of “lip tie,” where they learned about “lip tie,” whether they thought their child had “lip tie,” whether they had a child undergo “lip tie” division, and how concerned they would be if they thought their child had “lip tie.” Information on participant demographics and social media was collected.

**Results:**

Overall, 59.8% (193) of the 323 parents surveyed had heard of “lip tie”; of those, 17.1% (33) had a child that had undergone “lip tie” surgery. Most parents (91.2%, 176) thought “lip tie” caused breastfeeding problems. Roughly one‐quarter of parents (51 of 197 responses) rated their concern about “lip tie” as >8 of 10 on a Likert scale (mean, 5.7). The reported sources of “lip tie” information included lactation consultants (36.8%, 71), nurses (22.8%, 44), and pediatricians (31.6%, 61) as well as nonmedical sources, such as social media, family, and friends (68.4%, 132). Overall, 87% (282) of the 323 participants reported daily use of social media.

**Conclusion:**

Although many parents are concerned about “lip tie,” much of their information on this condition comes from nonmedical sources. Social media would be a valuable platform to provide accurate information on “lip tie.”

The maxillary frenulum, also called the superior labial frenulum or frenum, is a fold of the mucous membrane that connects the upper lip to the alveolar process in the midline of the maxilla.^1^ The maxillary frenulum arises as a posteruptive remnant of embryonic tectolabial bands^2^ and may provide stability to the lip.^3^ The structure comprises tight connective tissue interwoven with occasional skeletal muscle fibers.^4^ These muscle fibers originate from the orbicularis oris muscle of the upper lip^5^; there are no major vessels within the maxillary frenulum.^4^ The size of the frenulum and its insertion locations are variable among individuals.^6^

Although maxillary frenula are almost universally present and are considered normal structures, the point at which they can be classified as tethered, known colloquially as “lip tie” (LT), remains unclear.^7^ Short frenulum length and low insertion have been implicated in upper lip flange difficulty.^8^ Maxillary frenula have been described as abnormal when they interfere with an infant's ability to flange the upper lip and achieve a successful latch during breastfeeding.^9^ However, this system exhibits low reliability among relevant medical professionals.^10^ Although there are no standard measurements for the diagnosis of LT, parents increasingly bring their infants to otolaryngology clinics with requests for maxillary frenotomies (procedures for dividing frenula) to address breastfeeding difficulties.^11^ The purpose of this study was to determine what information parents have regarding LT, the sources of this information, and their level of concern about the condition.

## Methods

### Experimental Design

This cross‐sectional study was approved (STUDY00006408) by the University at Buffalo Institutional Review Board.

### Study Population

Parents of children between 0 and 12 years of age examined at an otolaryngology clinic between May and July 2022 were recruited for the study. Parents were approached consecutively by a research assistant, who gave them the paper survey to complete independently. Few parents refused to participate. The following parental demographic information was recorded: sex, age (categorized as <30, 30‐45, or >45 years), highest education attained, race and ethnicity, and occupation in the medical field, if applicable. All information was provided voluntarily in the clinic room behind a closed door, and no patient identifiers were collected.

### Survey

Parents were first asked if they had ever heard of LT. One parent per child was asked to complete the survey. Parents who reported having no knowledge of LT were only required to provide the following information: (i) the age of their youngest child, (ii) time they spent on social media (no time, <1 hour per month, <1 hour per week, <1 hour per day, or >1 hour per day), and (iii) favorite social media platform. Parents who reported that they had heard of LT were asked to indicate: (i) where they had learned about it, (ii) if they believed one of their children has or had LT, (iii) if their child had undergone surgery for LT, and (iv) their beliefs about the effects of LT. Possible effects that were listed on the survey included breastfeeding problems, bottle‐feeding problems, speech problems, reflux, and dental problems, among others. Additionally, parents were asked to rate how concerned they would be if they thought their child had LT; the rating was on a Likert scale of 1 (no concern) to 10 (greatest concern).

### Data Analysis

Information was tabulated and analyzed using SPSS 27 (2020; IBM Corp.). Categorical variables were summarized by frequency or percentage and associated 95% confidence limits. Continuous data were summarized by mean and 95% confidence intervals (CIs). Disease information and demographic variables, such as age and sex, were assessed by means of summary statistics. A Kolmogorov‐Smirnov test was performed to determine if the data for all variables were normally distributed. Simple descriptive statistics such as frequency, mean, standard deviation, minimum, and maximum were calculated for data that were normally distributed, otherwise, the median and interquartile range were calculated. Categorical and continuous variables were analyzed using *χ* ^2^ and Wilcoxon rank sum tests, respectively. Regression models were used when possible to estimate responses based on 1 or more predictor variables.

## Results

Surveys from a total of 323 parents were included in the study. The demographic characteristics of the parents are in Table 1. One hundred fourteen (35.3%) parents worked in the medical or dental field, among which the most common positions were nurses (28.1%, 32), nurse practitioners (5.3%, 6), and physician assistants (5.3%, 6). The mean age of the participants' youngest child was 2.40 years (95% CI, 2.39‐2.42 years).

**Table 1:** Demographic characteristics of the study population

Characteristic	No. (%) of parents (n = 323)
Sex	
Female	270 (83.6)
Male	53 (16.4)
Age, y	
<30	50 (15.5)
30‐45	262 (81.1)
>45	11 (3.4)
Highest education level	
Did not graduate high school	2 (0.6)
High school	29 (9.0)
Completed some college	51 (15.8)
College graduate	241 (74.6)
Race or ethnicity	
African American or black	14 (4.3)
Asian	8 (2.5)
Caucasian or white	288 (89.2)
Hispanic	10 (3.1)
Native American	2 (0.6)
Prefer not to say	1 (0.3)

**Figure 1:**
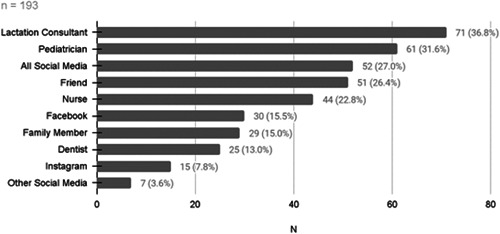
Where parents learned about “lip tie.”

More than half (59.8%, 193) of the parents who participated in the study had heard of LT, and 37.3% (72) believed their child had LT. The reported sources where the parents learned of LT are presented in Figure 1. Overall, 10.2% (33) of the study population had children who had undergone surgery to correct LT.

**Figure 2:**
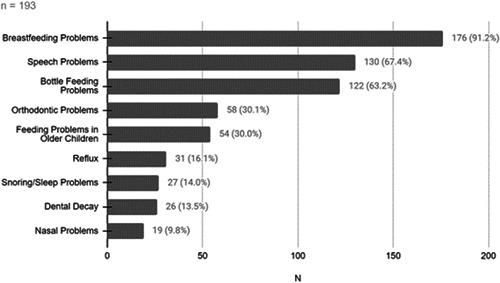
Problems attributed to “lip tie” according to parents.

**Figure 3:**
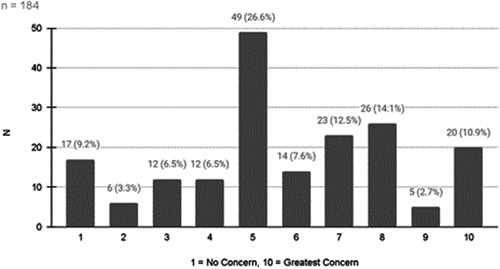
Parents rating of their concern about “lip tie.”

There was a wide range of problems that parents who had heard of LT attributed to the condition (Figure 2); on the survey, parents could select multiple concerns. They were also asked to rate how concerned they were about LT: 27.7% (51 of 184 responses) were very concerned (rating of 8 or higher) (Figure 3). The mean rating was 5.7 (95% CI, 5.37‐6.11).

**Figure 4:**
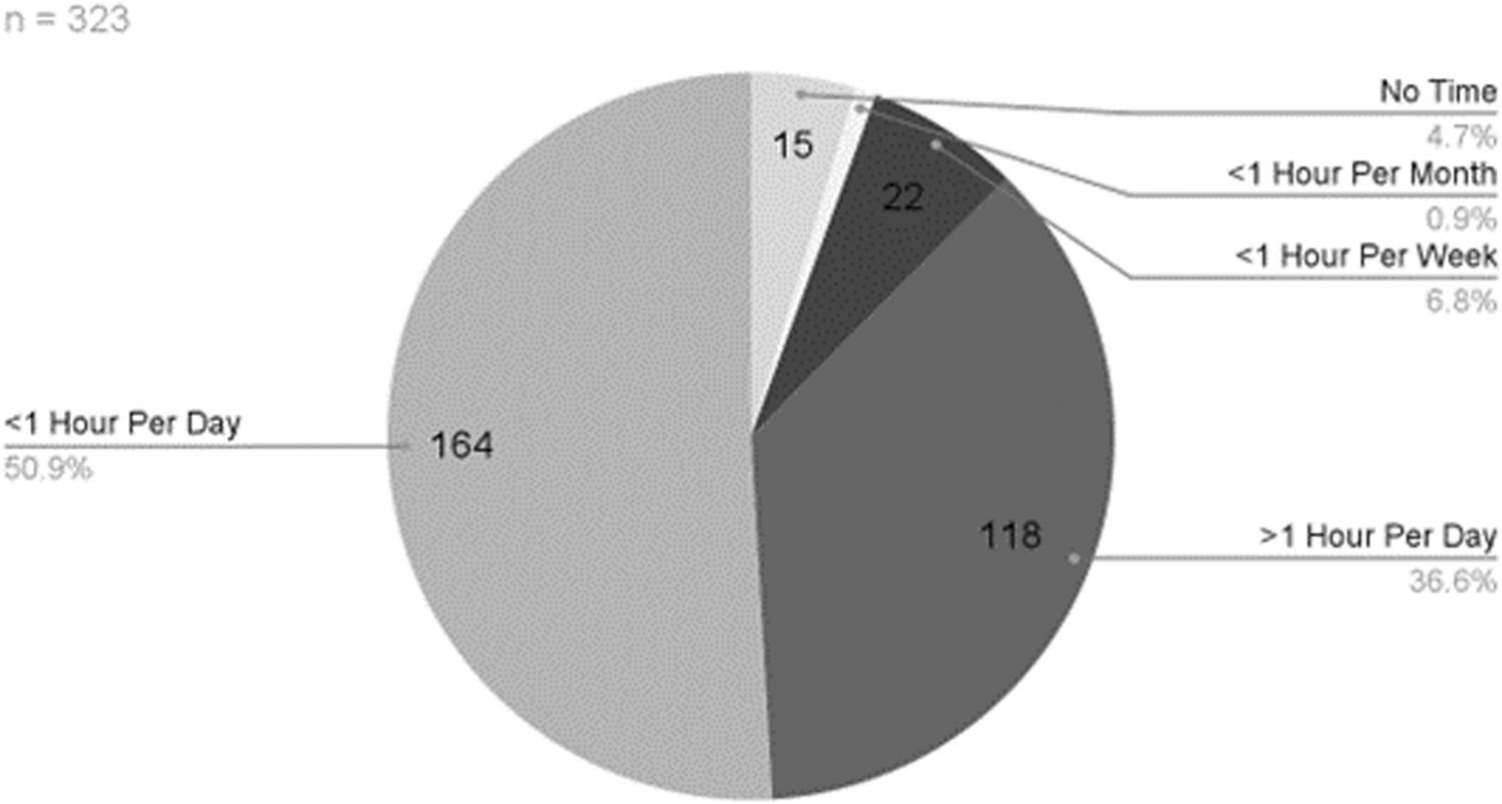
Time parents spent on social media.

When surveyed about the use of social media, most of the parents (87.3%, 282) reported using it daily; 36.5% (118) spent >1 hour per day using social media (Figure 4). The most popular social media platforms among parents were Facebook (40.9%, 132), Instagram (32.2%, 104), and TikTok (4.6%, 15).

## Discussion

There are mixed findings in the literature regarding the association between “lip tie” and latching or feeding issues, yet parents often present believing that maxillary frenotomy is necessary for their newborn's health.

### Overdiagnosis of LT

Recently, there has been a dramatic increase in the number of articles related to tongue‐tie, breastfeeding, and frenotomy.^12^ Concurrently, requests for maxillary frenotomy have also increased.^11^ The lack of consensus regarding the appearance, structure, function, significance, and treatment of LT has led to vastly differing opinions among parents and physicians from different specialties, including otolaryngology, pediatrics, dentistry, lactation counseling, and speech pathology.^13^ According to Naimer et al,^14^ “aside from the expert opinions that are unsubstantiated by research, hardly any data exists on the subject.” Because an infant's upper lip and frenulum are typically only examined in the context of a breastfeeding problem, parents may assume that the presence of any frenulum is abnormal,^10^ and clinicians may associate the variability of frenulum presentation with feeding difficulties.^3^ Selection bias may play a role in this generalization, as newborns with clinical symptoms, such as breastfeeding difficulty, are more likely to have their upper lip and maxillary frenulum examined.^10^ The lack of diagnostic criteria for LT enables clinician bias to influence frenotomy rates.^15^ Practitioners who perform LT release may overadvertise the beneficial effects for financial gain while ignoring the low morbidity associated with leaving the frenulum intact.^16^ Even in the absence of strong data to show a causal relationship between maxillary LT and these adverse effects,^7^ LT is seemingly overdiagnosed in many communities.^3^

### The Use of Social Media for Health Information

The majority of parents surveyed in our study who had heard of LT had received information from social media, family, and friends. Although the Internet serves as a major source of medical information, there is often a disconnect between professional recommendations and the information patients and parents read on nonmedical social media websites.^17^ Social media has the potential to provide parents with evidence‐based, user‐generated information, but it often distributes opinions that are not based on facts and may even be contradictory to professional recommendations.^18^ In a cross‐sectional survey of parents of children aged 0 to 18 years, 93% of respondents (240 of 258) had searched online for information about health or medical issues related to their child, and parents of young children were found to be most frequently exposed to child health information on social media.^19^ In a scoping review of literature pertaining to parental social media use by Frey et al,^18^ it was determined that parents are motivated to use social media as a health information source in order to seek or provide information, advice, support, and validation for medical decisions. In this review, 1 study found that parents trust other parents more than they do health care professionals when considering the health management needs of a child outside the consultation room.^18^, ^20^ Health information and knowledge based on lived experiences shared among parents were more readily accepted based on the belief that parents in the same predicament were inherently more trustworthy than health care providers.^21^

### Clinical Consequences of LT

In this study, the parents who reported prior knowledge of LT (59.8%, 193) attributed a wide variety of clinical conditions to LT. Primarily, parents felt that LT negatively impacted breastfeeding and dentition. In the literature, there are mixed findings on each of these presumed complications of LT.

#### Breastfeeding

Previous studies have suggested a link between LT and breastfeeding difficulties.^7^, ^16^, ^22^ In children with LT, restricted upper lip movement and an inability to flange the lip are implicated in feeding difficulties.^22^ For the past decade, there has been an increase in the promotion of exclusive breastfeeding in the United States.^23^ Consequently, the primary justification for maxillary frenotomy is to increase the ease of breastfeeding and improve maternal confidence.^10^ In a study by Patel et al,^24^ mothers showed increased satisfaction with breastfeeding (73%), less nipple pain (73%), and reduced noises during feeding (67%) after maxillary frenotomy. However, numerous other studies^7^, ^11^, ^25^ have not found a causal relationship between LT classification and breastfeeding difficulty. Although more prominent, lower‐lying frenulae are assumed to be more disruptive, anatomical classification of frenulae has not correlated with breastfeeding success.^7^ In a study by Stuebe et al,^25^ it was determined that infants who presented with LT did not have a higher incidence of disrupted lactation compared to the general population. In another investigation, scores on the LATCH scale, a systematic clinical tool used to assess maternal and infant variables affecting breastfeeding, were also not found to be correlated to maxillary frenulum grading.^11^ This implies that it may be beneficial to focus on breastfeeding technique education prior to exploring surgical options.^11^

Although maxillary and lingual frenula have been implicated in breastfeeding issues,^14^ few studies have assessed the impact of LT without concurrent ankyloglossia.^7^, ^22^ Evidence has supported the link between ankyloglossia and breastfeeding difficulty.^13^, ^26^ In studies that have suggested a link between breastfeeding difficulty and LT, the maxillary frenulum is often released at the same time as the lingual frenulum, preventing the quantification of the importance of LT release alone.^7^, ^14^ Compared to infants who present with concurrent LT and ankyloglossia, the number of infants presenting with LT alone is very small.^22^ Additionally, there is evidence that parents may be confusing the terms LT and “tongue tie.” In a study about parental perceptions of ankyloglossia and frenotomy outcomes on Twitter, 20% of the reviewed tweets mentioned LT.^17^

#### Dental Problems

There are numerous studies that have found an association between maxillary LT and maxillary midline dental diastema, which prevents contact between central incisors.^27^, ^28^ Early maxillary frenotomy may improve the appearance of diastema, therefore providing cosmetic benefits.^29^ Conversely, frenotomy poses an increased risk of scarring, which may make a diastema more likely in permanent dentition.^3^ Diastema between maxillary central incisors is common during primary and mixed dentition, and most close spontaneously as permanent dentition erupts.^30^ According to a 2020 clinical consensus statement, maxillary frenotomy in infants or children with primary dentition does not prevent the occurrence of midline dental diastema.^3^

Other dental issues, such as dental caries,^31^ plaque buildup,^6^, ^29^ and postorthodontic relapse^27^ have been implicated as clinical consequences of LT in children. In adults, LT may be related to periodontal disease, ill‐fitting dentures, and gingival recession,^32^ although no statistically significant correlation has been found with any of these conditions. Regarding periodontal disease, the presence of a severe diastema caused by a low‐lying maxillary frenulum attachment may lead to issues with oral hygiene and trapped food, but most frenulae are benign and do not typically have adverse dental outcomes.^33^

### Limitations

The limitations of our study primarily revolve around the location of data collection. Only 1 site was used, and it was centered in a homogenous, fairly affluent area with a low degree of ethnic diversity. Most of our survey respondents were college graduates, and almost 90% identified as Caucasian. We approached parents of children up to age 12 and did not specifically target families with newborn children, which may have impacted our results. Selection bias may have played a role in study participation, as it is possible that parents who were concerned about LT were more likely to agree to complete the survey. Additionally, there is a large dental practice within the same city that advertises laser LT division, which may influence whether parents seek information related to LT and whether they perceive LT as a condition to be corrected. In the future, this study should be repeated in a more ethnically and racially diverse area and across several data collection sites.

## Conclusion

A substantial proportion of parents are very concerned about LT, and much of their information on this condition comes from nonmedical sources. As the vast majority of parents reported using social media daily, otolaryngologists should consider social media platforms to promote evidence‐based information related to LT and frenotomy.

## Author Contributions

**Beatrice R. Bacon**, acquisition and interpretation of data, drafting and revision of the manuscript; **Michele M. Carr**, conception and design of the work, acquisition and interpretation of data, revision of the manuscript, and final approval of the version to be published.

## Disclosures

### Competing interests

The authors have no conflicts of interest to disclose.

### Funding source

None.
